# The complete chloroplast genome of *Medicago arabica* (Fabaceae)

**DOI:** 10.1080/23802359.2022.2067498

**Published:** 2022-04-23

**Authors:** Yingxue Jiao, Xiaofan He, Yuhua Shen, Yuehui Chao, Tiejun Zhang

**Affiliations:** a School of Grassland Science, Beijing Forestry University, Beijing, China; b College of Chemistry and Life Sciences, Chifeng University, Chifeng, China

**Keywords:** Chloroplast genome, *Medicago arabica*, Fabaceae

## Abstract

*Medicago arabica* (Linnaeus, 1762) Huds. is an important annual legume forage that grows in a wide range of climates, from subtropical to temperate. This study aimed to sequence the chloroplast genome of *M. arabica* and compare it with other legumes. In this study, we sequenced the entire chloroplast genome of *M. arabica*, which has 125,056 base pairs. The total GC content of the chloroplast genome of *M. arabica* was 34.4%. From the 110 unique genes of the circular genome, 30 tRNA genes, four rRNA genes, and 76 protein-coding genes were successfully annotated. A maximum likelihood (ML) tree was constructed using the model species and 17 species of the *Medicago* genus. *M. arabica* was shown to be phylogenetically closely related to *M. polymorpha*. The nucleotide diversity of the chloroplast genome may provide valuable molecular markers to study chloroplast, genetic breeding, and plant molecular evolution. These findings provide a solid foundation for future research on the molecular biology of the chloroplast.

*Medicago arabica* (Linnaeus, 1762) Huds., also known as spotted medic, is a flowering plant that belongs to the Fabaceae family. It is native to the Mediterranean basin and has since spread throughout the world, where it can be found growing on cliff tops and in different types of grasslands. *M. arabica* is one of the essential leguminous forages found worldwide, especially in subtropical and temperate climates (Nair et al. 2006).

According to the USDA, *M. arabica* has greater adaptability than other annual legumes and is used to improve soil properties and grazing productivity (Bialy et al. 2004). It has a symbiotic relationship with *Sinorhizobium medicae*, a bacterium capable of fixing nitrogen present in the soil. It is considered essential for pasture improvement because of its short vegetative period, flat or sub-flat stem type, sclerotized seeds, and ability to adapt to a wide range of environmental conditions (Tava et al. 2009). The aerial parts of *M. arabica* contain high concentrations of saponins that have solid fungicidal activity against several pathogenic fungi and have the potential to be developed as a natural source of fungicides (Saniewska et al. 2005).

The chloroplast is involved in photosynthesis, and the synthesis of key phytohormones is involved in defence responses and inter-organelle signaling (Bhattacharyya and Chakraborty 2018). This organelle also regulates starch storage, sugar synthesis, and critical cellular components, including amino acids, vitamins, pigments, lipids, and metabolic pathways for sulfur and nitrogen (Martin et al. 2013).

The chloroplast is a vital organelle in plants that contains genes and components specific to the chloroplast. In this evolutionary context, the arrangement of the chloroplast genome is remarkably conserved. The availability of complete chloroplast genome sequences can provide essential information for plant breeding, chloroplast genetic engineering, the development of valuable molecular markers, and phylogenetic analysis (Tao et al. 2017). The chloroplast genome of *M. arabica* will be a valuable source of genetic markers for determining evolutionary linkage as well as a robust platform for studying the evolution and genetic breeding of this crop.

The chloroplast genome sequence of *M. arabica* has not yet been reported, and further research into its chloroplast genomes is important and urgent. This study aimed to sequence and annotate the chloroplast genome of *M. arabica* and compare it with that of other legumes. In the present study, the chloroplast genome of *M. arabica* was sequenced and structurally characterized, providing an invaluable resource for future studies in the Fabaceae family, especially in the genetic evolution and genetic development of feed crops and other plant species.

Samples of *M*. *arabica* were collected from the Bajia Botanical Garden in Beijing, China (E116°29′, N40°03′). The seeds of *M*. *arabica* were deposited in the forage germplasm bank of the School of Grassland Science, Beijing Forestry University (Beijing, China; E116°29′, N40°03′). One specimen was deposited at the Herbarium of the School of Grassland Science, Beijing Forestry University (http://cxy.bjfu.edu.cn/, Tiejun Zhang, tiejunzhang@126.com) with the voucher number PI495212. Genomic DNA from post-emergence shoots was extracted using a DNA extraction kit from Shanghai Limin Industries Co., Ltd. (Shanghai, China). Sequencing was performed using the Illumina Novaseq PE150 platform (Illumina Inc., San Diego, USA), which generated 150 bp paired-end reads. The complete chloroplast genome was assembled from the cleaned reads using GetOrganelle v1.5 (Jin et al. 2020), which used the chloroplast genome of *Medicago truncatula* (GenBank accession number: NC 003119) as a reference. The chloroplast genome was annotated using CPGAVAS2 (Shi et al. 2019) and GeSeq (Tillich et al. 2017) and subsequently performed manually. The annotated chloroplast genome sequences are registered in GenBank with an accession number (MZ905469). The study of *M. arabica*, including collecting plant material, followed the standards established by the School of Grassland Science, Beijing Forestry University, and Chinese and international regulations. Field research adhered to Beijing legislation and followed all research protocols.

Our study revealed that the entire chloroplast genome of *M*. *arabica* is 125,056 base pairs long. The GC content of the entire chloroplast genome was 34.4%. The chloroplast genome of *M*. *arabica* consists of 110 different genes, including 76 protein-coding genes, 30 tRNA genes, and four rRNA genes. There are 30 genes encoding amino acid transfer proteins, 15 genes encoding light-harvesting structural proteins (PSII), 11 genes encoding NADH dehydrogenase proteins, and 11 genes encoding small subunit ribosomal proteins, which are found in the chloroplast genome of *M*. *arabica.*

To determine the phylogenetic relationships of *M. arabica*, the chloroplast genomes of 17 species of the *Medicago* genus, as well as *Melilotus albus* and *Trifolium repens* from sister groups of *Medicago* in Fabaceae as outgroup species, were downloaded from the GenBank database of the National Center for Biotechnology Information (NCBI). These sequences were aligned with the help of MAFFT v7 (Katoh et al. 2019). A maximum likelihood (ML) tree was also generated using the raxmlGUI 1.5 b programme (v8.2.10), which is based on the common protein-coding genes of 19 species and is based on the results of this study (Silvestro and Michalak 2012). The nucleotide sequences of 69 common genes were used to construct the ML tree. According to the results of the phylogenetic survey, *M. polymorpha* is closely related to *M. arabica* (Figure 1). This study provides valuable information for species identification and phylogenetic relationships within the Fabaceae family, mainly legume forage. It will provide a solid foundation for future research into the molecular biology of chloroplast, genetic breeding, and the molecular evolution of *M. arabica*.

**Figure 1.**
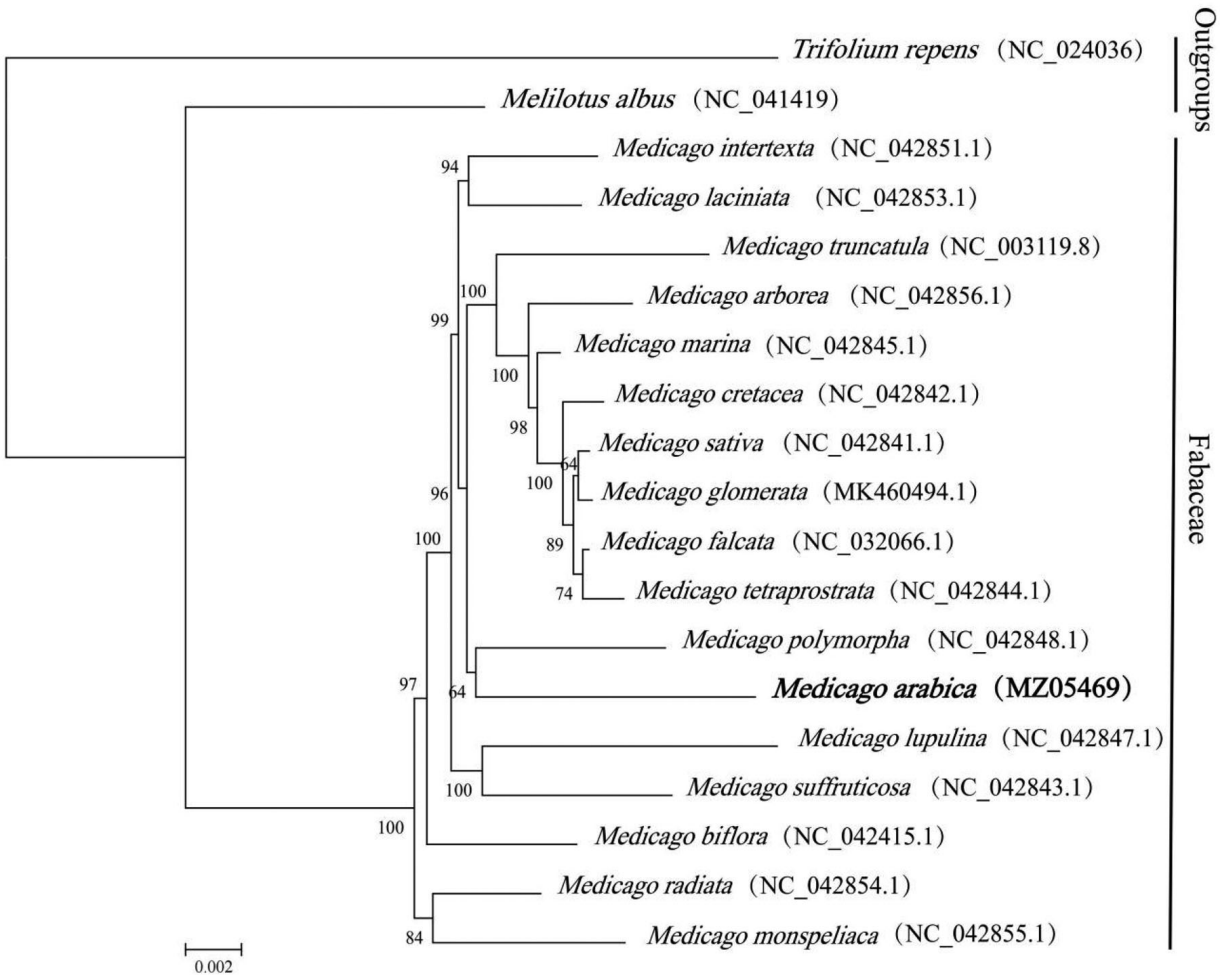
A phylogenetic tree was reconstructed using the maximum likelihood (ML) method based on shared protein-coding genes of 17 species of the *Medicago* genus. *Melilotus albus* and *Trifolium repens,* both members of sister groups of *Medicago* in Fabaceae, served as outgroups. The numbers above the lines represent ML bootstrap values (>70%).

## Data Availability

The genomic sequence data supporting the findings of this study are publicly available in NCBI GenBank (https://www.ncbi.nlm.nih.gov/) under the accession number MZ905469. The associated BioProject, SRA, and Bio-Sample numbers are PRJNA750257, SRR15275748, and SAMN20447317, respectively.
